# Treatment of Familial Hypercholesterolemia with Intracranial Xanthoma: Case Report

**DOI:** 10.3389/fsurg.2022.875422

**Published:** 2022-05-03

**Authors:** XiaoBo Kou, LunXin Liu, LiangXue Zhou

**Affiliations:** Department of Neurosurgery, West China Hospital, Si chuan University Chengdu, China

**Keywords:** xanthoma, familial hypercholesterolemia, intracranial, ezetimibe, retrosigmoid sinus approach

## Abstract

Intracranial xanthoma is a rare benign intracranial tumor. It often occurs in patients with hyperlipidemia. Intracranial xanthomas grow slowly, and clinical symptoms only appear when the mass compresses the surrounding tissues, so early diagnosis of the disease is difficult.

## Learning Point for Clinicians

Intracranial xanthoma is a rare benign intracranial tumor. It often occurs in patients with hyperlipidemia. Intracranial xanthomas grow slowly, and clinical symptoms only appear when the mass compresses the surrounding tissues, so early diagnosis of the disease is difficult.

## Case Report

**Figure 1:**
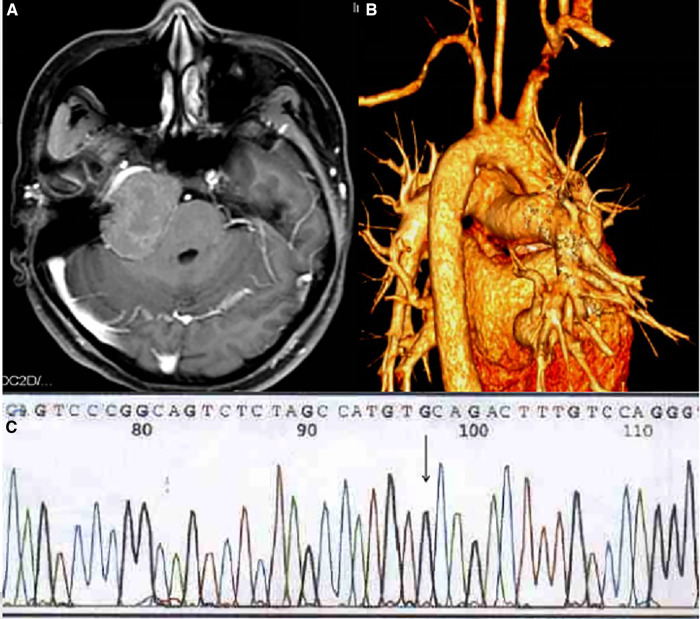
(**A**) MRI showd the skull base has long T1 and long T2 lesions on the right side of the middle straddle, with flair showing mixed signals; local bone destruction in the lesion. (**B**) Coronary CTA showed multiple mural thrombus with calcification in the proximal segment of the aortic arch and the three branches of the superior arch, moderate stenosis at the origin of the left common carotid artery. (**C**) T-G mutation at position 97 in exon 6 of LDL-R gene (arrow).

**Figure 2:**
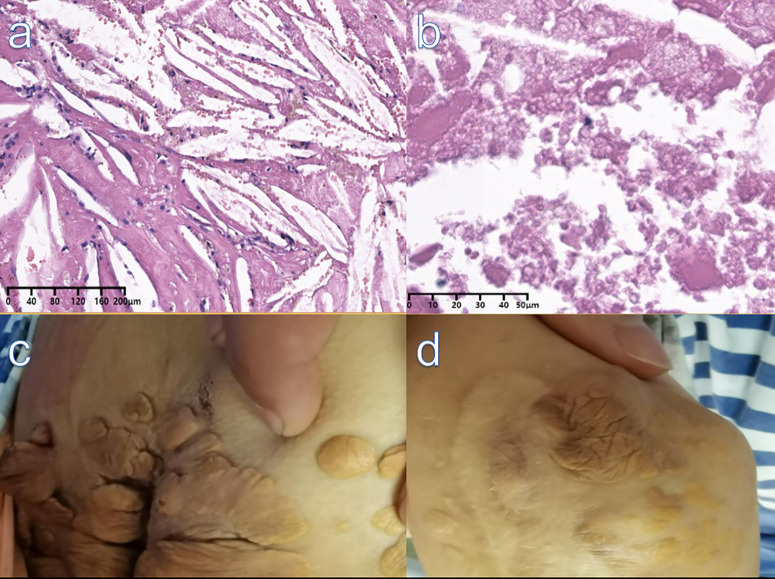
(**A**) (H&E,X10), (**B**) (H&E,X40): Pathology showed fibrous tissue hyperplasia with abundant cholesterol fissures, Foam cell aggregation with necrosis and calcification. (**C**) Xanthomas on the skin of the buttocks. (**D)** Scar and residual xanthoma after excision of left elbow mass.

A 25-year-old female complained of right-sided facial paralysis and hearing loss for 4 years, and an intracranial mass was found for 1 week. The patient had a history of left elbow xanthoma resection (**Figure 2D**) and familial hypercholesterolemia; the patient’s LDL-R genetic test showed a T-G mutation at position 97 in exon 6 (**Figure 1C**), supporting the diagnosis of homozygous familial hypercholesterolemia (HoFH); her parents had no relevant medical history, and her brother was diagnosed with heterozygous familial hypercholesterolemia (HeFH). Positive sign: the right eye was incompletely closed, the right frontal lines and nasolabial fold became shallow, right ear hearing loss was observed, and multiple masses were seen in the skin of the right popliteal fossa and buttocks (**Figure 2C**), and excision of a left elbow mass (**Figure 2D**). MRI showed the following: right middle posterior skull base mixed density mass (size of 4.6 cm × 4.1 cm × 3.7 cm), focal bone destruction (**Figure 1A**); Coronary CTA showed: Moderate stenosis at the origin of the left common carotid artery(**Figure 1B**); cholesterol (TC): 14.9 mmol/L, low-density lipoprotein (L-DL): 12.87 mmol/L. After admission, ezetimibe was taken orally to reduce blood lipids. All tumors were removed by a surgray throwed retrosigmoid sinus approach under electrophysiological monitoring. Postoperative pathology showed xanthoma (**Figure 2A,B**).

## Discussion

The formation of xanthoma may be due to vascular adventitial cells ingesting lipoproteins and macrophage aggregates. Following lipoprotein exudation, these cells transform into vacuolized foam cells, within which serum lipoproteins are metabolized. Subsequent aggregation of cholesteryl esters, cholesterol, and phospholipids leads to the formation of xanthomas. According to whether companion has hyperlipoproteinemia, it can be divided into two kinds: hyperlipoproteinemia xanthoma and nonhyperlipoproteinemia xanthoma (1). Here, we present a rare case of HoFH complicated with intracranial xanthoma.

The pathogenesis of xanthoma is still controversial. At present, there are two theories about the formation mechanism of xanthoma. One theory is that local trauma or hemorrhage leakage causes lipids to enter peripheral tissues from blood vessels. The lipids accumulate in the cells and are swallowed by macrophages to form foamy macrophages and extracellular cholesterol. Crystals enter the gap and induce inflammation in macrophages and fibroblasts. The other theory is that abnormal elevation of blood lipids leads to an inflammatory response in undifferentiated mesenchymal cells. After a series of inflammatory reactions, they are phagocytosed by macrophages to form foam cells. This article reports that the formation of intracranial xanthomas may be related to the deposition of cholesterol crystals or the blockage of the adjacent mastoid air cells, leading to local tissue hypoxia and blood cell necrosis (2, 3).

It has been reported in the literature that intracranial xanthomas mostly occur in the temporal occipital region (4). The MR manifestations are mixed T1 and T2 signals, with flaky fat signal shadows, which are distinguished from diseases such as lipomas, cholesteatoma, and skull tumors (5). Small xanthomas may have no clinical manifestations when there is no pressure on the surroundings. Imaging examination can help us make an early diagnosis and determine the range of lesions and their adjacent relationships (6).

Xanthoma is a rare benign intracranial tumor that needs to be differentiated from meningioma, cholesteatoma, lipoma, and other intracranial tumors. Early diagnosis of HoFH and effective drug treatment may reduce the incidence of xanthoma. For xanthomas with clinical manifestations, complete or partial resection of the tumor is required to relieve symptoms, and drugs are given for etiological treatment to prevent recurrence after surgery. In the future, gene therapy is worth looking forward to, and it may fundamentally solve this problem.

## Data Availability

The original contributions presented in the study are included in the article/Supplementary Material, further inquiries can be directed to the corresponding author/s.
